# Comparison of two peroxidases with high potential for biotechnology applications – HRP *vs.* APEX2

**DOI:** 10.1016/j.csbj.2024.01.001

**Published:** 2024-01-12

**Authors:** Sanja Škulj, Matej Kožić, Antun Barišić, Aitor Vega, Xevi Biarnés, Ivo Piantanida, Ivan Barisic, Branimir Bertoša

**Affiliations:** a Department of Chemistry, Faculty of Science, University of Zagreb, Horvatovac 102a, Zagreb HR-10000, Croatia; b Institute of Physiology, Pathophysiology and Biophysics, Department of Biomedical Sciences, University of Veterinary Medicine Vienna, 1210 Vienna, Austria; c Laboratory of Biochemistry, Institut Químic de Sarrià, Universitat Ramon Llull, Via Augusta 390, 08017 Barcelona, Spain; d Division of Organic Chemistry & Biochemistry, Ruđer Bošković Institute, Bijenička Cesta 54, 10 000 Zagreb, Croatia; e Molecular Diagnostics, Center for Health and Bioresources, AIT Austrian Institute of Technology GmbH, Giefinggasse 4, Vienna 1210, Austria; f Eko Refugium, Crno Vrelo 2, Slunj 47240, Croatia

**Keywords:** Peroxidase, Glycosylation, Horseradish peroxidase, Ascorbate peroxidase, HRP, APX, Enzyme engineering, Molecular dynamics simulations

## Abstract

Peroxidases are essential elements in many biotechnological applications. An especially interesting concept involves split enzymes, where the enzyme is separated into two smaller and inactive proteins that can dimerize into a fully active enzyme. Such split forms were developed for the horseradish peroxidase (HRP) and ascorbate peroxidase (APX) already. Both peroxidases have a high potential for biotechnology applications. In the present study, we performed biophysical comparisons of these two peroxidases and their split analogues. The active site availability is similar for all four structures. The split enzymes are comparable in stability with their native analogues, meaning that they can be used for further biotechnology applications. Also, the tertiary structures of the two peroxidases are similar. However, differences that might help in choosing one system over another for biotechnology applications were noticed. The main difference between the two systems is glycosylation which is not present in the case of APX/sAPEX2, while it has a high impact on the HRP/sHRP stability. Further differences are calcium ions and cysteine bridges that are present only in the case of HRP/sHRP. Finally, computational results identified sAPEX2 as the systems with the smallest structural variations during molecular dynamics simulations showing its dominant stability comparing to other simulated proteins. Taken all together, the sAPEX2 system has a high potential for biotechnological applications due to the lack of glycans and cysteines, as well as due to high stability.

## Graphical Abstract

**Figure:**
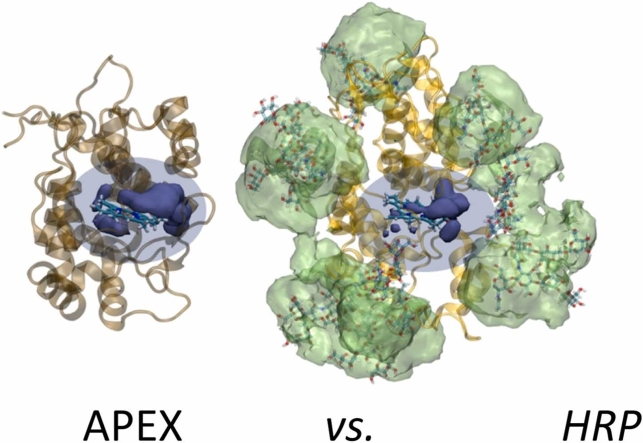

## 1 Introduction

Peroxidases are enzymes that catalyze the H_2_O_2_-dependent oxidation of a wide array of small-molecules [1], [2]. One of the best investigated is the horseradish peroxidase (HRP) [3], [4], [5], [6], [7], [8]. It was originally isolated from the roots of horseradish plants and is extensively used in biochemistry and molecular biology. HRP is a heme-containing glycoprotein that catalyzes the oxidation of various substrates using hydrogen peroxide as an electron acceptor, leading to the formation of reactive intermediates. It is often employed as a marker in immunohistochemistry and as reporter molecule in enzyme-linked immunosorbent assays (ELISA). HRP's ability to produce a colored product upon reaction with its substrate makes it valuable in visualizing target molecules and for diagnostic applications. For most applications, addition molecules such as streptavidin are covalently attached to HRP to enable controlled molecular interactions to *e.g.*, antibodies or biotinylated nucleic acids. Non-glycosylated variants expressed in *E.coli* are also interesting due to their altered surface properties and potentially easier expression and purification but are structurally less stable [9]. The effect of the glycosylation on the structural and dynamical properties of wild type and split HRP was analyzed in detail in a previous study [10]. Another popular peroxidase is APX derived from soybean plants. It is not glycosylated which makes its purification significantly easier. APX is especially popular for proximity labeling techniques in cell biology and cellular imaging. In general, peroxidase-catalyzed reactions have a high potential for biotechnology applications and have become a valuable tool to decipher *in vivo* protein-protein interactions by protein engineering [11], [12], [13], [14], [15]. A very interesting and highly specific approach to analyze protein-protein *in vivo* is based on the concept of split peroxidases. The enzymes can be split into two parts, which are further engineered by introducing several mutations stabilizing the protein structure in the split form. Each part is then fused to two different proteins for which their reciprocal interaction aims to be interrogated. In the split form, the two divided parts are inactive, but if they meet in solution, they will produce a reconstituted peroxidase domain with recovered activity [16], [17], [18], [19], [20], [21], [22], [23]. This feature provides huge potential for a wide range of biotechnology applications because wrong positive signals and background noise can be avoided [24]. A common substrate to monitor the redox reaction of HRP is the chromogenic substrate ABTS (2,2′-azinobis [3-ethylbenzothiazoline-6-sulfonic acid]-diammonium salt) [7], [25]. Accessibility of the reporting substrate to the active site needs to be warranted when designing split forms of peroxidases for these applications.

**Fig. 1:**
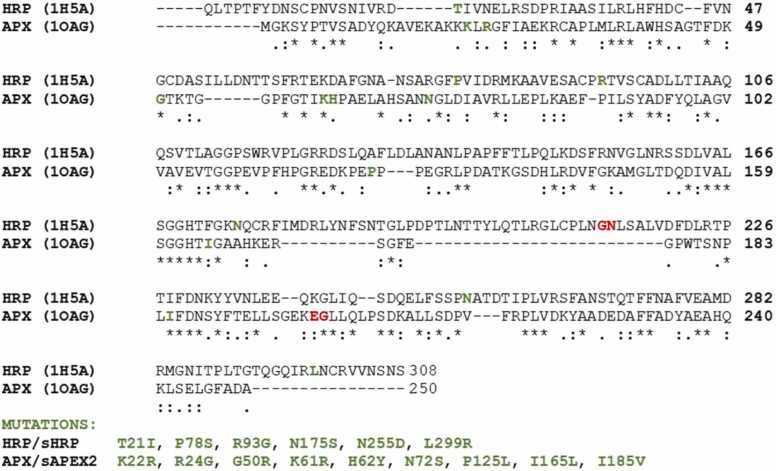
Alignment of HRP and APX primary sequences. sHRP and sAPEX2 mutated residue positions are depicted in green and split position in red. (For interpretation of the references to colour in this figure legend, the reader is referred to the web version of this article.)

**Fig. 2:**
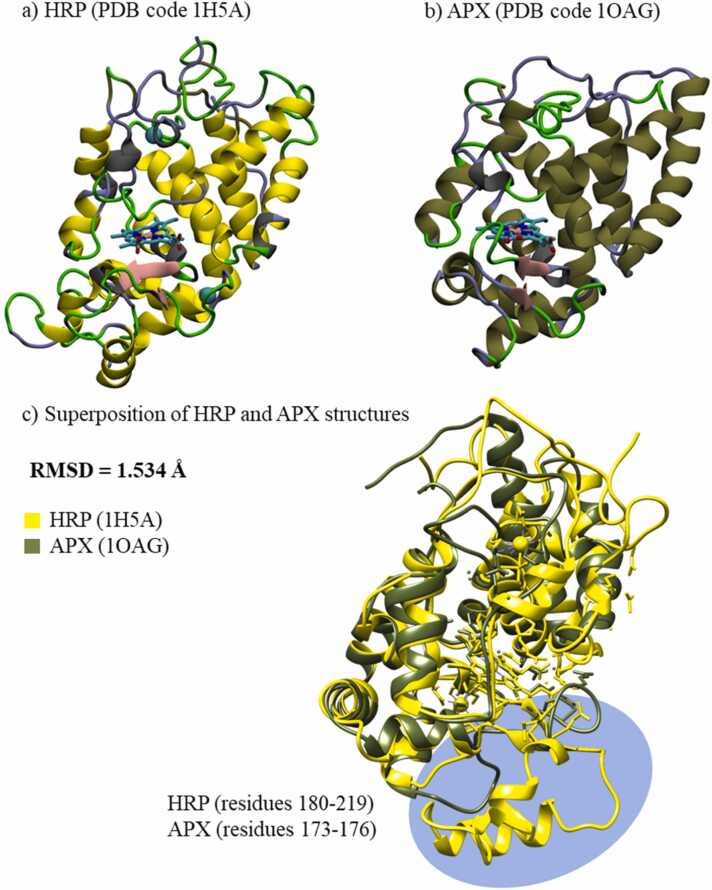
Tertiary (crystal) structures of: a) HRP and b) APX peroxidases colored by secondary structures. Heme group is presented as sticks and calcium ions as VDW spheres. c) Superimposed HRP and APX structures. (For interpretation of the references to colour in this figure legend, the reader is referred to the web version of this article.)

In this paper, we computationally analyzed the two peroxidases HRP and APX and their split forms [3], [4], [5], [6], [7], [8]. Based on the Enzyme Commission Number (ECN), both enzymes belong to the peroxidases class – EC 1.11.1. Based on substrate specificity, APX belongs to L-ascorbate peroxidase (ECN:1.11.1.11), whereas HRP to a phenolic radical donor (ECN:1.11.1.7). The sequence identity between HRP [8] and APX [4] is 28% (Fig. 1). With 250 amino acids, APX has shorter protein sequence than HRP (308 amino acids). Both enzymes have similar tertiary structures (Fig. 2) and catalyse the oxidation of similar organic substrates.

Despite many similarities, these two peroxidases have also important structural differences (Table 1 and Fig. S1); HRP has 9 potential *N*-glycosylation sites following the pattern Asn-X-Thr/Ser whereas APX does not possess patterns for *N*-glycosylation [26], [27], [28], [29], [30]. Another important difference is the presence of four disulphide bonds and two calcium ions in HRP which are not present in APX.

**Table 1:** Structural features of APX and HRP enzymes.

enzyme	heme group	glycosylation (Asn-X-Thr/Ser)	calcium ions	disulphide bonds	mutations in engineered form
**HRP** (1H5A)	✓ (His 170)	✓ (9)	✓ (2)	✓ (4)	✓ (6)
**APX** (1OAG)	✓ (His174)	✗ (0)	✗ (0)	✗ (0)	✓ (9)

This paper presents the comparison of both wild type forms and split engineered forms of the two peroxidases, HRP and APX. The split HRP engineered structure (sHRP) has 6 stabilizing mutations, and it is split between residues 213–214 [16], while the split APX structure (sAPEX2) has 9 stabilizing mutations and it is split between residues 200–201 (Fig. S1 in SI) [17]. The structural and dynamical properties of these two enzymes, the solvent and ligand accessibility to the active site, the catalytic site topology, and substrate binding were examined for both the wild type and split forms. In the first studies using split peroxidases, only *in vivo* experiments were presented avoiding the demanding expression and purification of the fusion proteins. Very recently, Heo *et al*. presented a promising approach to exploit the split enzyme concept also for *in vitro* experiments [31]. The aim of this work is to develop a deeper understanding of mechanistic behavior of the split peroxidases and pave the way for synthetic approaches to attach proteins and DNA to the split enzymes. These engineered enzymes have huge potential for diagnostic applications.

## 2 Results

### 2.1 Structural and dynamical properties

**Fig. 3:**
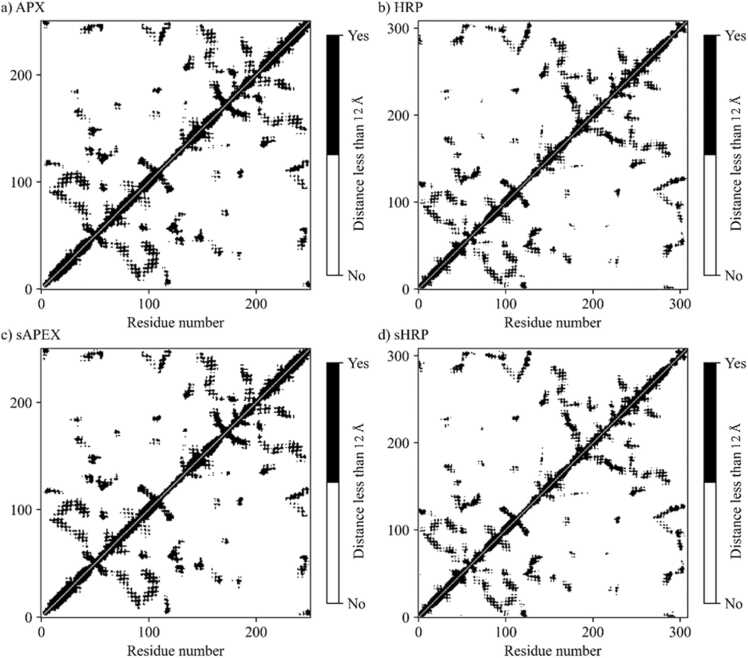
Pairwise contact map of Euclidean distances between the Cα atoms that are less than 12 Å in an equilibrated protein structure of: a) APX, b) HRP, c) sAPEX2 and d) sHRP.

**Fig. 4:**
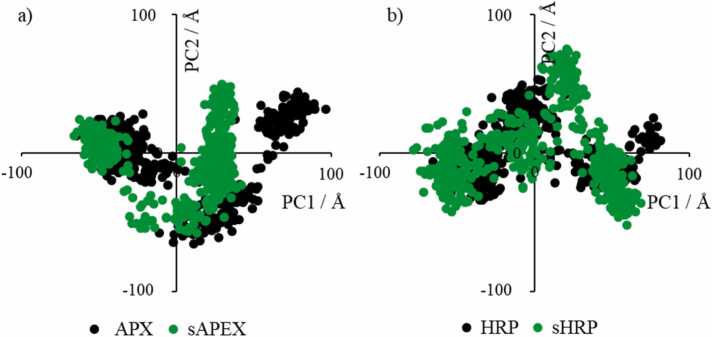
Principal component analysis (PCA) of a) APX/sAPEX2 and b) HRP/sHRP performed on the simulated protein atom trajectories (see Methods).

Two enzymes, horseradish peroxidase (HRP) and ascorbate peroxidase (APX), have similar structural properties. Despite only 28% sequence identity between the two enzymes, the 3D structures of the two peroxidases are alike having Root Mean Square Deviation (RMSD) of 1.534 Å. Secondary structure is consisting mostly of alpha helices with one antiparallel beta sheet with two strands and heme placed inside helices (Fig. 2). The major difference between the structures is in the HRP loop with the short alpha helix (residues 180 - 219), which is absent from the APX structure (residues 173 - 176) (Fig. 2c). The contact maps (Fig. 3, Fig. S2in SI) confirm structural similarities between the four systems; pairwise contact and heat maps have similar patterns of interactions among amino acids in tertiary structures of proteins. All four systems are stable during the 500 ns of molecular dynamics (MD) simulations, as showed by RMSD values below 2.5 Å along the three replica simulated trajectories (Fig. S3 and S5 in SI). Mutations and introduced split truncation in sHRP and sAPEX2 do not affect on overall protein stability. Fluctuations along the protein chain are similar in all variants, as observed from root mean square fluctuations (RMSF) calculation along the three replica trajectories (Fig. S4, S6 and S7 in SI). Main structural variations of the systems were also investigated through Principal Component Analysis (PCA). This kind of analysis is based on identifying the spatial directions along which each protein residue presents higher displacements with respect to the average. Comparison of the patterns observed in the plot allows recognizing differences in the type of protein motions between systems. Space occupied by the first two principal components (PC1 and PC2), which account for majority of the overall protein motion (over 50%) have analogous and alike space shape for both proteins (Fig. 4, Fig. S8). PCA shows similarity between HRP and sHRP, suggesting the truncation of HRP into sHRP does not affect protein dynamics. The dynamics of APX is also similar to HRP. Interestingly, dynamics of sAPEX2 along the PC1 is different (Fig. 4a, Fig. S8b), identifying sAPEX2 as the system with the smallest structural variations among all four studied systems.

**Fig. 5:**
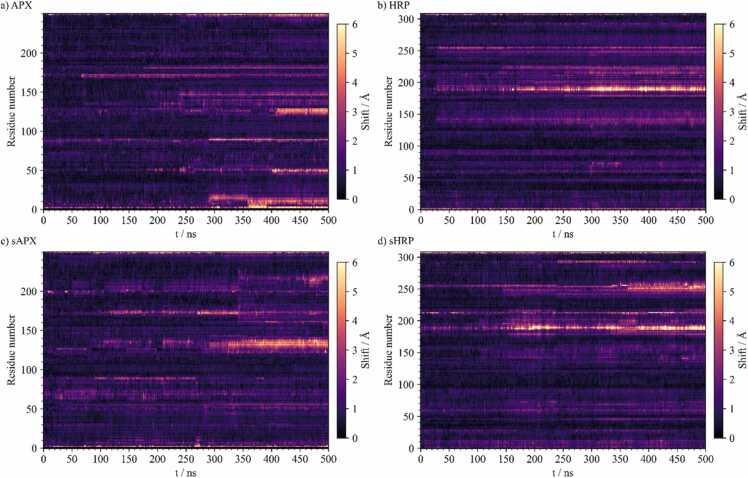
Trajectory maps for: a) APX, b) HRP, c) sAPEX2 and d) sHRP proteins. Shifts are calculated as the Euclidean distance between the center of mass of a residue’s backbone in each frame of a simulation and its starting position of a protein aligned during the trajectories.

Residue displacements during the simulation, relative to the protein, were computed and plotted using heat-maps (Fig. 5), referred to as trajectory maps. This visualizes the relative protein chain motion along the simulated trajectories, showing the courses of simulations. Trajectory map analysis showed that sHRP has a higher number of more intense bands which correspond to conformational events of bigger magnitude compared to HRP (Figs. 5b and 5d). On the other hand, trajectory maps for sAPEX2 showed fewer intense bands. Notably, near the end of the APX and sAPEX2 simulations, trajectory maps differ in the amount and intensity of bands corresponding to residues ∼0–18, ∼45–53, and ∼99 (Figs. 5a and 5c). Average backbone relative displacements of sAPEX2 during the last 200 ns of simulation was calculated to be 1.24 ± 0.08 Å, compared to higher average displacements during the last 200 ns of APX simulation 1.31 ± 0.13 Å. Thus, trajectory maps also confirm that sAPEX2 yields a structure more stable than the wild type APX simulation.

### 2.2 Active site accessibility

**Fig. 6:**
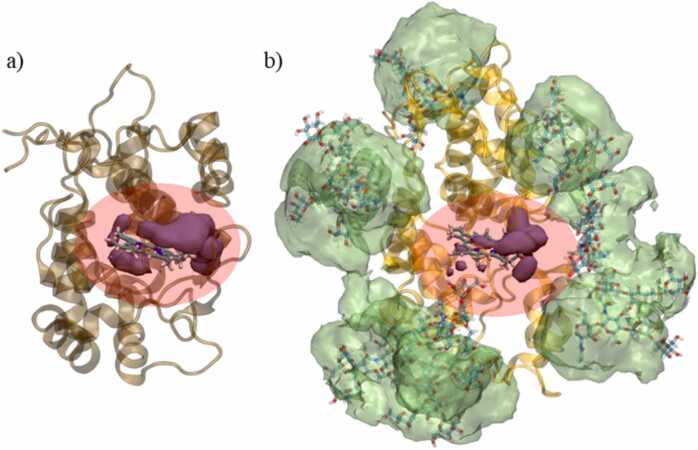
Average volume occupied by water molecules (purple volume) around 3 Åof heme group during the 500 ns of MD simulations of: a) APX and b) HRP. Active sites of both enzymes are marked with a red transparent ellipse. In transparent green colour, the average volumes of Man_5_GlcNAc_2_ are presented during the 500 ns of MD simulations. (For interpretation of the references to colour in this figure legend, the reader is referred to the web version of this article.)

An active site accessibility is necessary for an adequate exchange of substrates and products during the peroxidase catalytic cycle. This was monitored for all four systems during the 500 ns of MD simulations. In case of both studied peroxidases, the active site entrance/exit is placed between “opened” alpha coils and it is accessible to the solvent (Fig. 6). Sterically, it is the only place of the protein’s surface easily accessible to substrate. Notably, glycans, which stabilize the structure of HRP and sHRP [22], do not sterically block the active site entrance/exit (Fig. 6b). In order to quantify the active site accessibility, average number of water molecules within a radius of 5 Å around the heme prosthetic group is calculated in 500 ns long MD simulations (Table 2, Fig. S9 in SI).

**Table 2:** Average number and standard deviation of water molecules within 5 Å from the heme group, His42 and Fe^2+^ during 500 ns of MD simulation.

	Fe^2+^	His42	Heme
**APX**	2 ± 1	8 ± 2	24 ± 3
**sAPEX2**	3 ± 1	8 ± 2	28 ± 5
**HRP**	2 ± 1	6 ± 2	16 ± 2
**sHRP**	2 ± 1	6 ± 1	15 ± 3

The results show that the position of water molecules in the active site is similar for all four studied systems. In average, the number of water molecules at the active site entrance is ∼60% higher for the APX/sAPEX2 than for HRP/sHRP (Fig. 6, Table 2). This is in agreement with calculations of the cavity area of both crystal structures, where APX (PDB:1OAG) has a larger volume of 946 Å [3] compared to HRP (PDB:1H5A) of 568 Å [3]. The catalytic amino acid His42 follows the same trend where more water molecules around histidine are present in APX and sAPEX. However, when we look at water molecules around Fe^2+^ in heme, there are always in average two water molecules in all proteins. Moreover, the ratio of water molecules around His 42/heme and Fe/heme in all four systems is comparable during simulation (Fig. S10). The number of water molecules around the wider region of the active site cavity and around the narrower essential regions of the catalytic center is similar in all systems, respectively.

### 2.3 Catalytic site of peroxidases

**Fig. 7:**
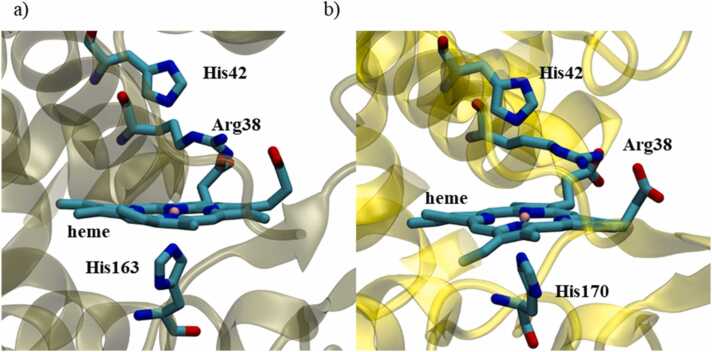
Catalytic centre of: a) APX and b) HRP protein. Snapshots are taken on middle structure from the most populated cluster in last 200 ns of MD simulation. Hydrogen atoms are hidden for clarity.

The catalytic sites of both peroxidases share similar features. The prosthetic heme group is bound to the protein through a coordinate bond from the iron ion to the proximal histidine (His163 in APX/sAPEX2, His170 in HRP/sHRP). The key catalytic residues His42 and Arg38 which are involved in the formation of Compound I are at identical positions in the wild type and split form of APX/sAPEX2, as well as in HRP/sHRP system (Fig. 7). The above-mentioned catalytic residues are in average less fluctuating than the rest of the protein (Table 3, Fig. 5 and Fig. S4 in SI). Mutations and cut site in engineered sAPEX2 and sHRP are not close to the catalytic site and do not affect its topology which is preserved during 500 ns of simulations (Fig. S1 in SI).

**Table 3:** Average fluctuations (RMSF) of the key catalytic amino acids during 500 ns of MD simulations.

RMSF / Å	APX	sAPEX2	HRP	sHRP
Arg38	0.6	0.5	0.4	0.5
His42	0.5	0.5	0.4	0.5

### 2.4 ABTS binding site in HRP

Experimentally determined structures of HRP and APX in complex with small ligands are available in the protein data bank. However, there is no crystal structure of these proteins with the large ligand ABTS. The binding mode of ABTS to the catalytic site of HRP was studied using docking analysis followed by MD simulation (see methods). The computational results were compared to the available crystal structure of the APX complex with its natural substrate ascorbate (PDB code 1OAF) [4].

**Fig. 8:**
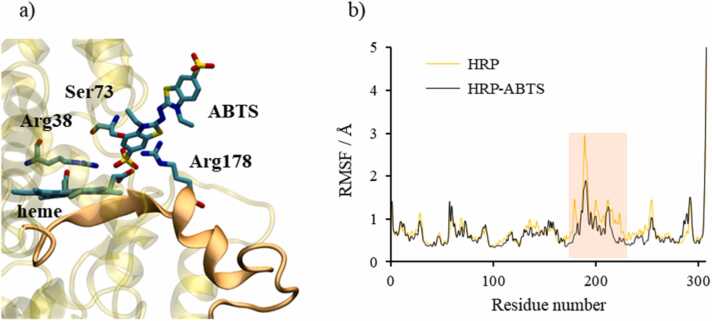
Substrate (ABTS) binding in the catalytic site of HRP and protein stabilization. a) Equilibrated ternary complex at the catalytic site of HRP. Secondary structures with the most pronounced decrease in fluctuations, residues 170–225, are coloured in orange. ABTS substrate and amino acids Arg38, Arg178, and Ser73 are presented in sticks. The rest of the protein is in transparent yellow cartoon representation. Snapshot is taken using the representative structure of the most populated cluster of the last 200 ns of MD simulation. Hydrogen atoms are hidden for clarity. b) Protein fluctuations (RMSF) during HRP (orange) and HRP-ABTS complex (black) MD simulations. (For interpretation of the references to colour in this figure legend, the reader is referred to the web version of this article.)

The sterically and energetically most favourable binding mode of ABTS was selected among the ensemble of computed HRP-ABTS complexes. The selected protein-ligand complex was subjected to a 500 ns of all-atom MD simulation. The simulation confirmed the stability of the binding mode since ABTS remained stable in complex with heme during the whole simulation (Fig. S11 in SI). Substrate binding affected the protein’s stability that became even more stable compared to the simulation of the protein without substrate (Fig. S12 in SI). Moreover, fluctuations along the protein decrease in the presence of ABTS ligand with pronounced reductions of fluctuations for the residues 170–225 that are directly linked to the ABTS ligand. This region forms an antiparallel β-sheet next to the heme and one part of α-helix extended to a β-sheet (Fig. 8). The rigidity of this region is a consequence of the binding of the ABTS substrate to the active site. Interestingly, this fragment of the protein is stabilized by direct protein-substrate interactions with the ABTS ligand. Arg178, Arg38, and Ser73 (Fig. 8) are important for ABTS stabilization in the catalytic site. The mentioned interactions were stable through the whole MD simulation (Fig. S13 in SI). Arg178 (Arg172 in APX) in the active site is well conserved and very important for substrate binding in both catalytic sites. In general, it is well known when protein is complexed with a substrate, its thermostability increases. This is correlated with a decrease in conformational flexibility [32].

**Fig. 9:**
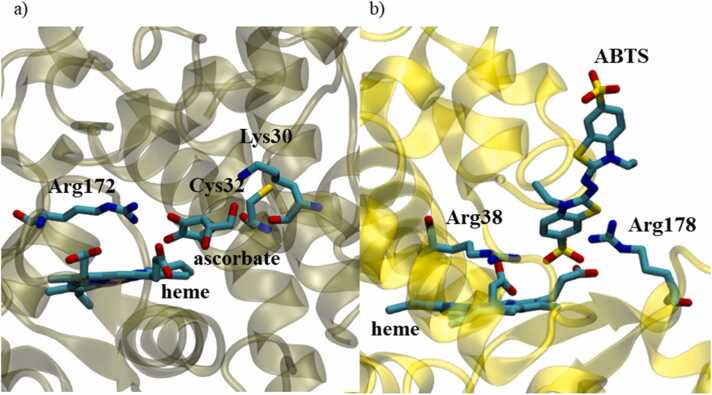
Substrate binding into the catalytic site of: a) APX - crystal structure (PDB ID: 1OAF) of APX-ascorbate and b) HRP – obtained by ABTS docking study followed by 500 ns of all-atom MD simulation. Snapshot is taken on middle structure from the most populated cluster in last 200 ns of MD simulation. Hydrogen atoms are hidden for clarity.

The computationally obtained HRP-ABTS complex was compared to the APX-ascorbate crystal structure [4] to highlight the differences in the ligand binding pocket between both enzymes. The predicted model of ABTS-HRP binding is compatible with the binding of ascorbate to APX (Fig. 9). In both structures, the substrate is oriented in accordance with previously presented results of the active site accessibility (see Section 2. Active site accessibility). Although highly similar, the topology of the two binding sites is not identical, since both ligands differ. The position of ascorbate in complex with APX is close to heme-6-propionate, while in the HRP-ABTS complex, the substrate location is next to heme-7-propionate (Fig. 9). Different residues at equivalent positions of the catalytic site mediate protein-ligand interactions. For instance, Cys32 and Lys30 interacting with ascorbate in APX are Arg38 and Ser73 interacting with ABTS in HRP. Notably, the well-conserved arginine in the primary sequence of the active site (Arg172 in APX, Arg178 in HRP, Fig. 1) is highly important for substrate binding in both catalytic sites. Notably, the well-conserved arginine in the primary sequence of the active site (Arg172 in APX, Arg178 in HRP, Fig. 1) is highly important for substrate binding in both catalytic sites. Surprisingly, this arginine belongs to area of pronounced structural difference between the two proteins, residues 180 - 219 in HRP and residues 173 - 176 in APX (Fig. 2c).

## 3 Discussion

The comparison of two peroxidase structures, each as wild type and in split form, was conducted with the goal of identifying possible differences that would prefer one system to another for biotechnology applications [4], [8], [16], [17]. The results of the study show that all four systems, HRP/sHRP, APX/sAPEX2, have similar contact maps, similar catalytic site appearance, and similar active site accessibility. Therefore, both enzymes, HRP/sHRP and APX/sAPEX2, are highly similar in terms of overall protein fold and catalytic site. Mentioned similarities are necessary because it is known that both proteins have highly similar mechanism of functioning [12], [13], [15], [33]. However, the differences such as glycosylation, disulfide bridges and calcium ions present only in HRP, induce changes in protein structure rigidity and can influence the application of split variants of these enzymes. Since HRP/sHRP is glycosylated, it is bigger in size – 44 kDa compering to 28 kDa of non-glycosylated APX/sAPEX2 [12]. In our previously published data, glycosylation plays stabilizing role in HRP/sHRP structures [10]. This is confirmed in recent circular dichroism spectroscopy measurements in tandem with MD simulations [34].

Computational results show that introduced mutations and cut site in both enzymes do not have significant influence on catalytic site appearance and active site accessibility and that stability of both proteins is remained. These results are in agreement with newest experimental results validating established study that sHRP engineered structure is stable and fully functional when two subunits are reconstituted [31]. However, sHRP structure is inactive in reducing environment due to the instability of disulfide bridge and calcium ions, while sAPEX2 remains active in the reducing environment like the cellular cytosol [33], [35]. From the other side, when it comes to resistance to denaturation by organic solvent treatments, disulphide bond present in HRP/sHRP improves stability compared to APEX. In this case, HRP/sHRP retain very high catalytic activity on human cell lines in methanol, the activity of full-length APX was completely abolished upon exposure to methanol. [36].

Comparison of the results obtained from the ABTS-HRP docking study followed by MD simulations with available APX-ascorbate crystal structure showed similar substrate binding modes of both peroxidases, albeit having different binding pockets. The APEX2 split variant is identified as the system with the smallest structural variations during MD simulations that, consequently, indicates its structural stability. Together with the lack of glycans, disulfide bridge and calcium ions, this may have the advantage of a better reconstitution of the active peroxidase domain from the independent split fragments when fused to different interacting proteins. Taken all together, small advantage for biotechnology applications for protein-protein interactions identification could be given to sAPEX2.

## 4 Methods

### 4.1 System preparation

**Fig. 10:**
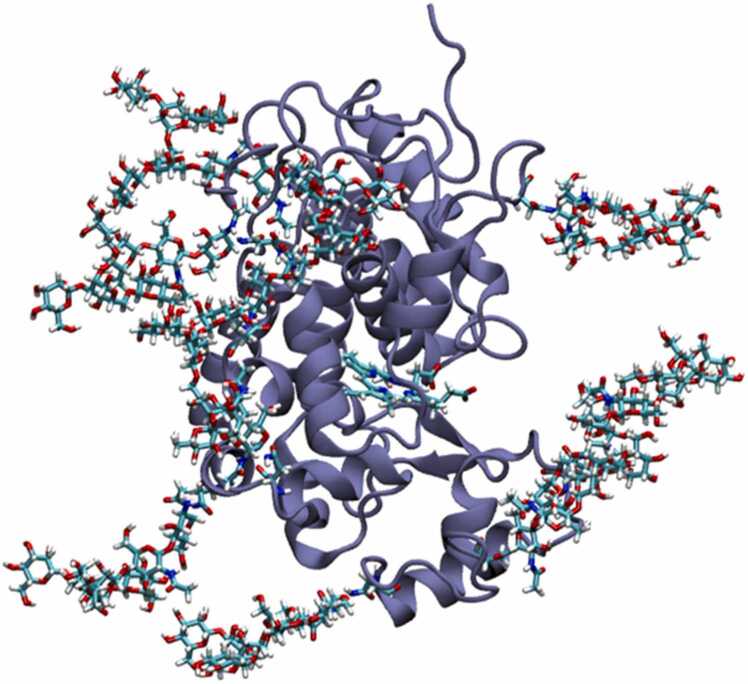
HRP with Man_5_GlcNAc glycosylation type. Presented structure was obtained after 500 ns of MD simulation.

Starting from the available crystal structures of APX – Ascorbate peroxidase from soybean cytosol of *Glycine max* (PDB code 1OAG) [4] and HRP – Horseradish peroxidase C1A from *Armoracia rusticana* (PDB code 1H5A) [8], four systems were prepared for molecular dynamics simulations: wild type (i) APX and (ii) HRP, and engineered split forms (iii) sAPEX2 and (iv) sHRP. ABTS coordinates were taken from crystal structure complex with FAD-dependent oxidoreductase (PDB code 7AA2) [37]. sHRP was prepared from HRP structure by introducing mutations of 6 amino acids (T21I, P78S, R93G, N175S, N255D, L299R) and split between residues 213–214 [16]. sAPEX2 structure was prepared from APX structure by introducing mutations of 9 residues (K22R, R24G, G50R, K61R, H62Y, N72S, P125L, I165L, I185V) and split between residues 200–201 [17]. Asparagine amino acids which follow the pattern Asn–X–Thr/Ser (X is any amino acid residue other than proline and aspartic acid) were *N*-glycosylated with Man_5_GlcNAc (Fig. 10) glycosylation type. 9 glycosylated asparagine residues in HRP are: 13, 57, 158, 186, 198, 214, 255, 268, 286 and 8 glycosylated asparagine residues in sHRP are residues 13, 57, 158, 186, 198, 214, 268, 286. Glycosylation is done using protocol described in previously published data [10].

Hydrogen atoms were added using CHARMM-GUI [38], [39], [40] in a way that the side chains of all arginines and lysines were positively charged, histidines (with hydrogen on epsilon nitrogen – HIE) and cysteines were prepared in their neutral form while side chains of glutamates and aspartates were deprotonated and negatively charged. Protonation states were checked by H+ + and PROPKA servers and decided for pH= 7 [41], [42]. Four disulphide bonds (Cys11-Cys91, Cys44-Cys49, Cys177-Cys209, and Cys97-Cys301) were added in case of HRP and three (Cys11-Cys91, Cys44-Cys49, and Cys177-Cys209) in case of sHRP. The bond between Fe^2+^ from the heme cofactor and His170 in case of HRP/sHRP or His163 in case of APX/sAPEX2 was defined. Calculations of the cavity area were calculated using online server CAVER [43].

### 4.2 Parametrisation

The CHARMM36m [44] force field was used for parametrization of protein structure, glycans, heme, ions and ABTS substrate. Solvation effects were simulated using periodic boundary conditions (PBC) with a cubic box filled with the TIP3P model of water molecules. The distance between the solute and the edge of the box was at least 20 Å. The size of a rectangular box depends on the system, but it was on average around 100 Å x 100 Åx 100 Å. Chloride ions were added to neutralize the systems.

### 4.3 Molecular dynamics simulations

Prior to molecular dynamics (MD) simulations, all systems were energy minimized (geometry optimized) in 1000 cycles and then equilibrated for 10 ns in the equilibration process provided by the CHARMM-GUI solution builder module, with different restraints being subsequently applied [38]. The production phase of MD simulations lasted for 500 ns for each system with a time step of 2 fs and the LINCS algorithm to keep all bonds constrained. MD simulations were performed in the isobaric-isothermal ensemble (NPT) employing periodic boundary conditions (PBC) in all directions at T = 300 K, which was maintained *via* a Nosé -Hoover thermostat [45] with a coupling constant of 1.0 ps^—1^. Pressure was set to 1.013 bar and was controlled with a semi-isotropic Parrinello-Rahman barostat [46] with a time constant for pressure coupling of 5 ps^—1^. Long range electrostatics were calculated using the particle-mesh Ewald (PME) [47], [48] method with real space Coulomb interactions cut off at 12 Åusing a Fourier spacing of 1.2 Åand the Verlet cut-off scheme.

All MD simulations were conducted in three independent simulations using GROMACS 2018.6 software package [49]. Analyses of trajectories were performed using GROMACS analysis tools and VMD program [50].

### 4.4 Custom made scripts

For quantifying the active site accessibility, a VMD script was written for counting the number of water molecules around parts of interests such that it counts “OH2″ type entities in a desired radius around desired parts. The results were visualized in Python using Matplotlib library. For creating contact maps a Python script was written that calculates Euclidean distances between each individual residue individually from every other, with a use of libraries Pandas [51], Numpy [52], and Matplotlib 3.4.3 [53], in Spyder 5 scientific integrated development environment for Python 3.9 [54], [55]. Structures analysed in that manner were starting equilibrated structures of MD simulations. Trajectory maps were obtained using TrajMap.py [56], an open-source Python based program fully available *via* a GitHub repository.

### 4.5 Docking calculations

Docking study was performed using and ensemble of 10 structures of HRP protein gathered from the 500 ns long MD simulation at fixed time intervals, as well as on the equilibrated structure of HRP protein (structure obtained after geometry optimization and short 10 ns equilibration). The structure of the ligand ABTS (2,2′-azinobis [3-ethylbenzothiazoline-6-sulfonic acid]-diammonium salt) was directly taken from the protein data bank (PDB code 7AA2, crystal structure of ABTS in complex with oxidoreductase) [37]. Protein and ligand parametrization was performed with AutoDock Tools4 [57] using the AutoDock 4.2 atom typing. The grid size was set to a cubic box of 20 Å x 20 Å x 20 Å) centered at the heme prostetic group. Docking calculations were performed using AutoDock Vina 1.1.2 [57] using default settings. Reference binding modes were selected based on a dual criterion: low energy binding modes and catalytically competent orientations of the ligand (Fig. S14). The starting structure for MD simulation of the HRP-ABTS complex was selected from the docking calculations performed on the equilibrated HRP protein structure.

## Funding information

This study was funded by the European Union’s Horizon 2020 Research and Innovation Programme under grant agreement No 952110, project Marilia.

## CRediT authorship contribution statement

**Sanja Škulj**: Investigation, Conceptualization, Writing – original draft, Writing – review & editing, Formal analysis, Visualization. **Matej Kožić**: Investigation, Formal analysis, Writing – review & editing. **Antun Barišić**: Investigation, Formal analysis. **Aitor Vega-Sánchez**: Investigation, Formal analysis. **Xevi Biarnés**: Supervision, Methodology, Writing – review & editing. **Ivo Piantanida**: Writing – review & editing; Project administration. **Ivan Barišić**: Funding acquisition, Project administration, Writing – review & editing. **Branimir Bertoša**: Conceptualization, Methodology, Supervision, Writing – original draft, Writing – review & editing, Project administration.

## Declaration of Competing Interest

The authors declare that they have no known competing financial interests or personal relationships that could have appeared to influence the work reported in this paper.
