# Supplementary material for: Comparison of two peroxidases with high potential for biotechnology applications – HRP vs. APEX2
Source: Comput Struct Biotechnol J. 2024 Jan 12;23:742–51. doi: 10.1016/j.csbj.2024.01.001 (PMC10828542; doi:10.1016/j.csbj.2024.01.001)
Supplement: Supplementary file 1 — Supplementary material [file mmc1.docx]

**Supporting Information**

Comparison of Two Peroxidases with High Potential for Biotechnology Applications – HRP *vs.* APEX

Sanja Škulj^1,2^, Matej Kožić^1^, Antun Barišić^1^, Aitor Vega Sánchez^3^, Xevi Biarnés^3^, Ivo Piantanida^4^, Ivan Barišić^5,6^, Branimir Bertoša^1, *^

1. Department of Chemistry, Faculty of Science, University of Zagreb, Horvatovac 102a, Zagreb HR-10000, Croatia.
2. Institute of Physiology, Pathophysiology and Biophysics, Department of Biomedical Sciences, University of Veterinary Medicine Vienna, 1210 Vienna, Austria
3. Laboratory of Biochemistry, Institut Químic de Sarrià, Universitat Ramon Llull, Via Augusta 390, 08017 Barcelona, Spain
4. Division of Organic Chemistry & Biochemistry, Ruđer Bošković Institute, Bijenička cesta 54, 10 000 Zagreb, Croatia
5. Molecular Diagnostics, Center for Health and Bioresources, AIT Austrian Institute of Technology GmbH, Giefinggasse 4, Vienna 1210, Austria.
6. Eko Refugium, Crno Vrelo 2, Slunj 47240, Croatia.

* Corresponding author:

Prof. Branimir Bertoša

Department of Chemistry, Faculty of Science, University of Zagreb, Horvatovac 102a, HR-10000 Zagreb, Croatia

Phone: +385 1 4606 132

e-mail: bbertosa@chem.pmf.hr


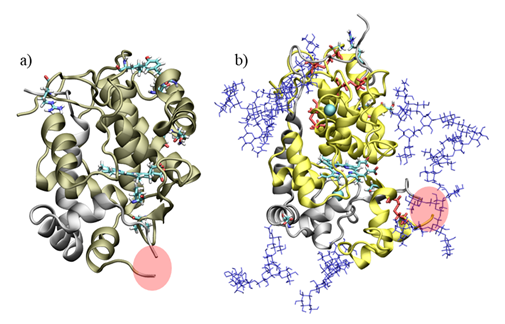


Figure S1: Mutated split structures of engineered enzymes: a) sAPEX and b) sHRP. Larger subunit of split form is colored in brown for sAPEX (left, residues 1-199) and in yellow for sHRP (right, residues 1 to 213). Smaller subunit of split form is colored in iceblue (residues 200-250 in sAPEX and 214-308 in sHRP). Mutated amino acids and heme are presented in licorice. Disulphide bonds are presented in red licorice. HRP calcium ions are presented in VDW representation. Split positions are shaded in transparent red color. Man_5_GlcNAc_2_ glycans of sHRP are presented in blue sticks.


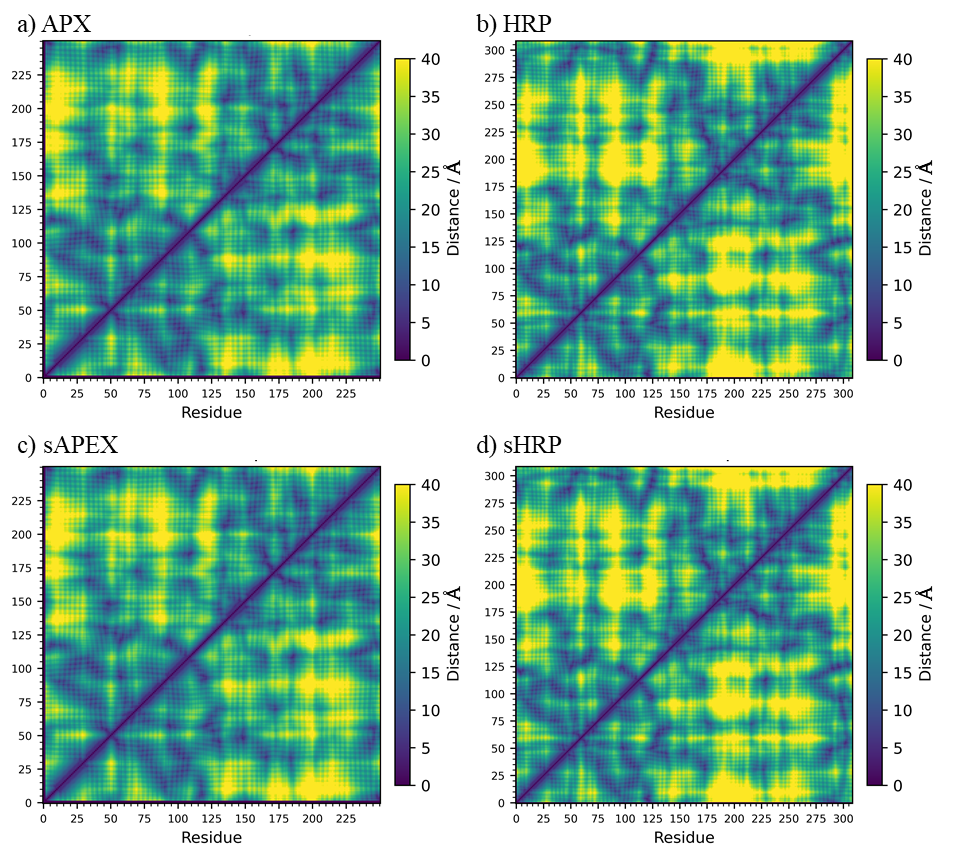


Figure S2: Pairwise distance maps for: a) APX, b) HRP, c) sAPEX, and d) sHRP protein obtained from equilibrated protein structures. Red and blue areas represent the maxima and minima of the color-scale range, indicating the clipping of values.


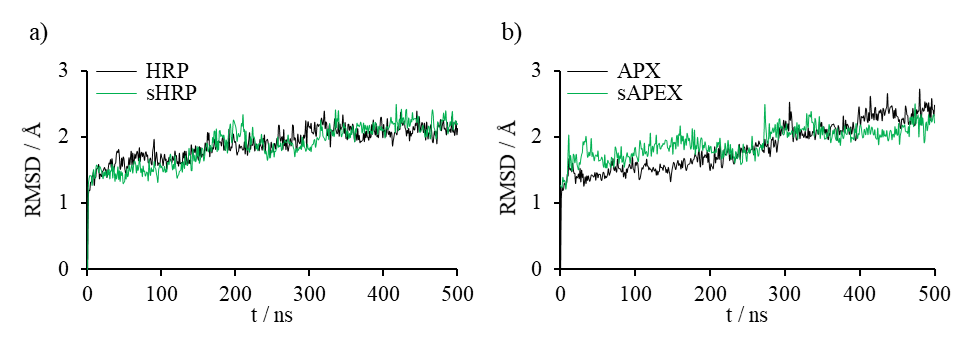


Figure S3: Root mean square deviations (RMSD) calculated during 500 ns molecular dynamics simulations for: a) HRP/sHRP and b) APX/sAPEX2. All protein atoms were considered in calculations.


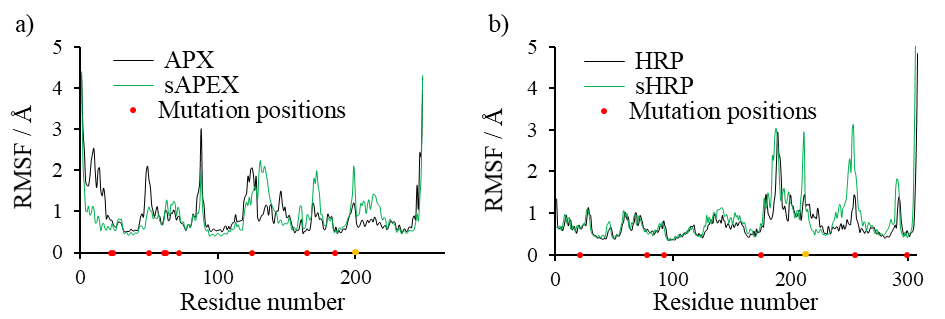


Figure S4: Fluctuations (RMSF) of: a) APX (black) and sAPEX (green); b) HRP (black) and sHRP (green). Split positions of sAPEX (200-201) and sHRP (213-214) are marked with yellow dots. Mutated residues in split forms are marked with red dots. The backbone carbon atoms (Cα atom) of every amino acid were considered in the calculation.


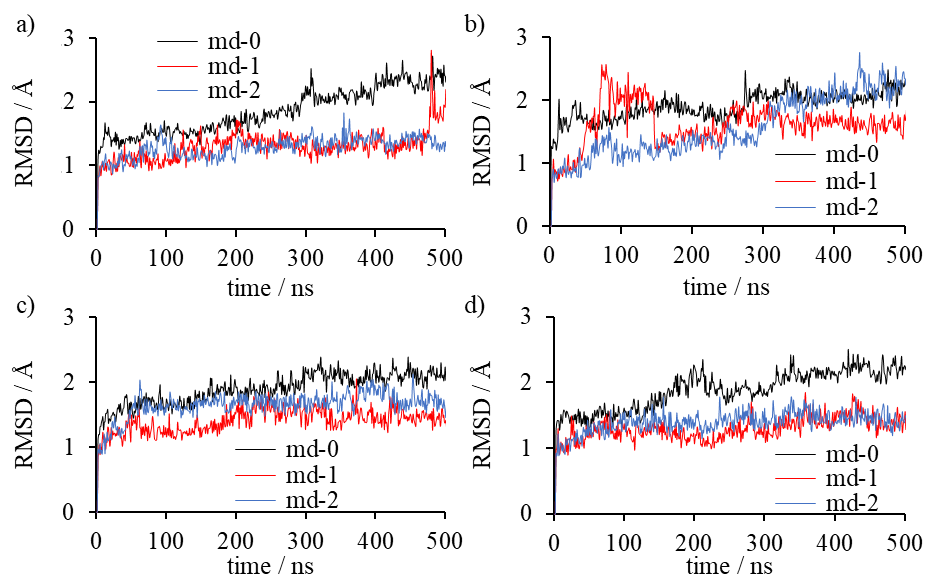


Figure S5: Root mean square deviations (RMSD) during molecular dynamics simulations of each system containing three replicas for: a) HRP, b) sHRP, c) APX and d) sAPEX2.


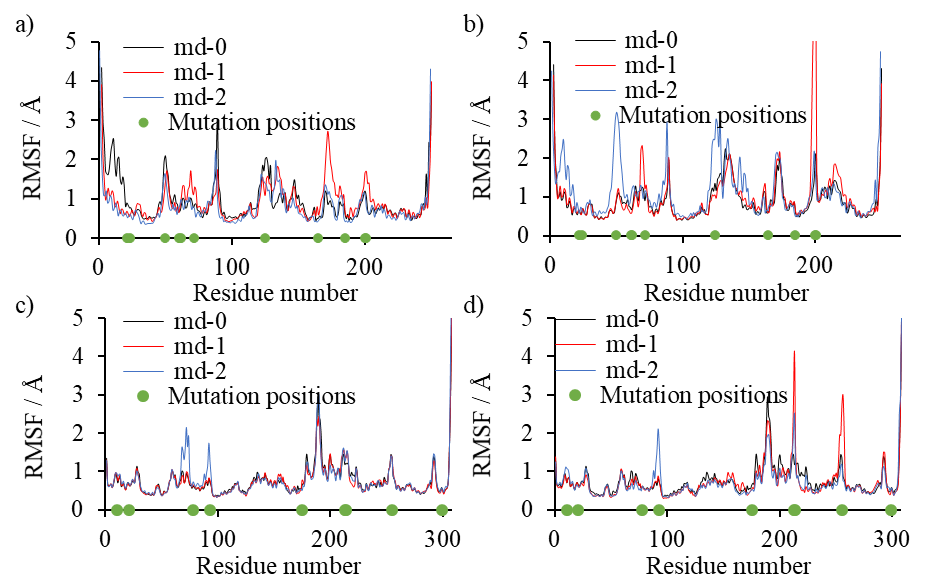


Figure S6: Fluctuations (RMSF) during 500 ns molecular dynamics simulations of each system containing three replicas for: a) APX and b) sAPEX2 c) HRP and d) sHRP. Mutated residues in split forms are marked with green dots. The backbone carbon atoms (Cα atom) of every amino acid were considered in the calculation. Reference structure is the starting structure.


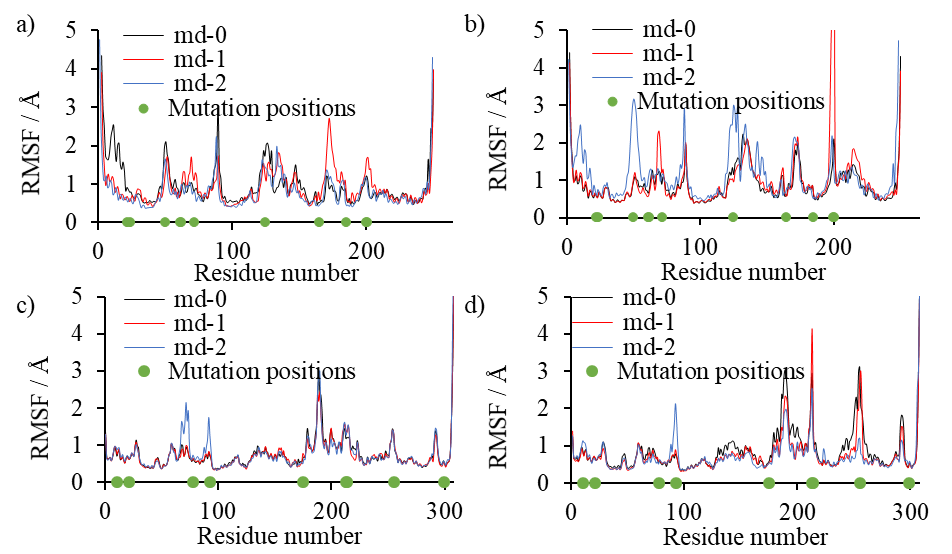


Figure S7: Fluctuations (RMSF) during 500 ns molecular dynamics simulations of each system containing three replicas for: a) HRP, b) sHRP, c) APX and d) sAPEX2. Mutated residues in split forms are marked with green dots. The backbone carbon atoms (Cα atom) of every amino acid were considered in the calculation. Reference structure is the most populated structure from cluster analysis.


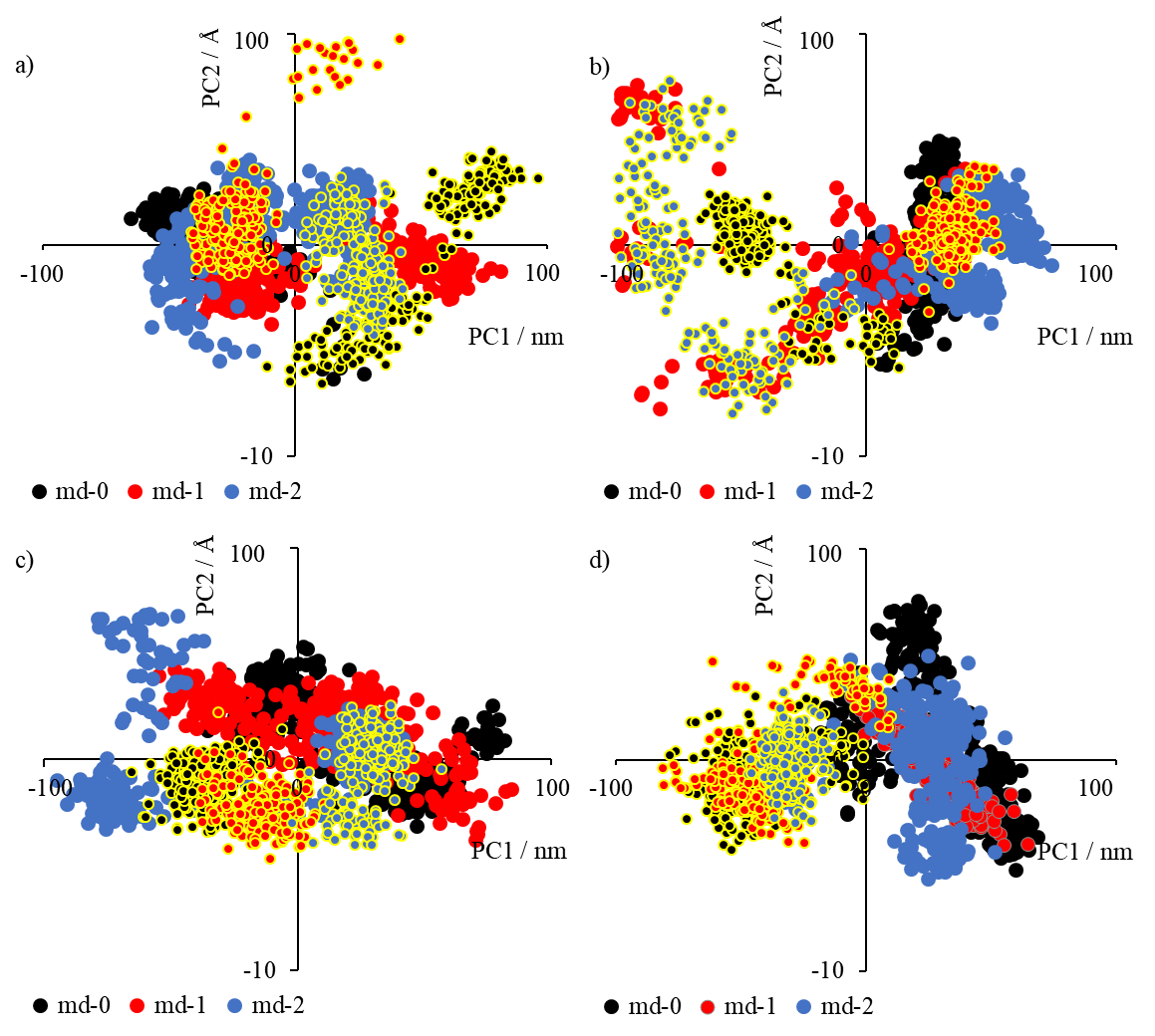


Figure S8: Principal component analysis (PCA) during 500 ns molecular dynamics simulations of each system containing three replicas for: a) APX, b) sAPEX2, c) HRP and d) sHRP performed on the simulated protein atom trajectories. Last 200 ns points are circled in yellow.


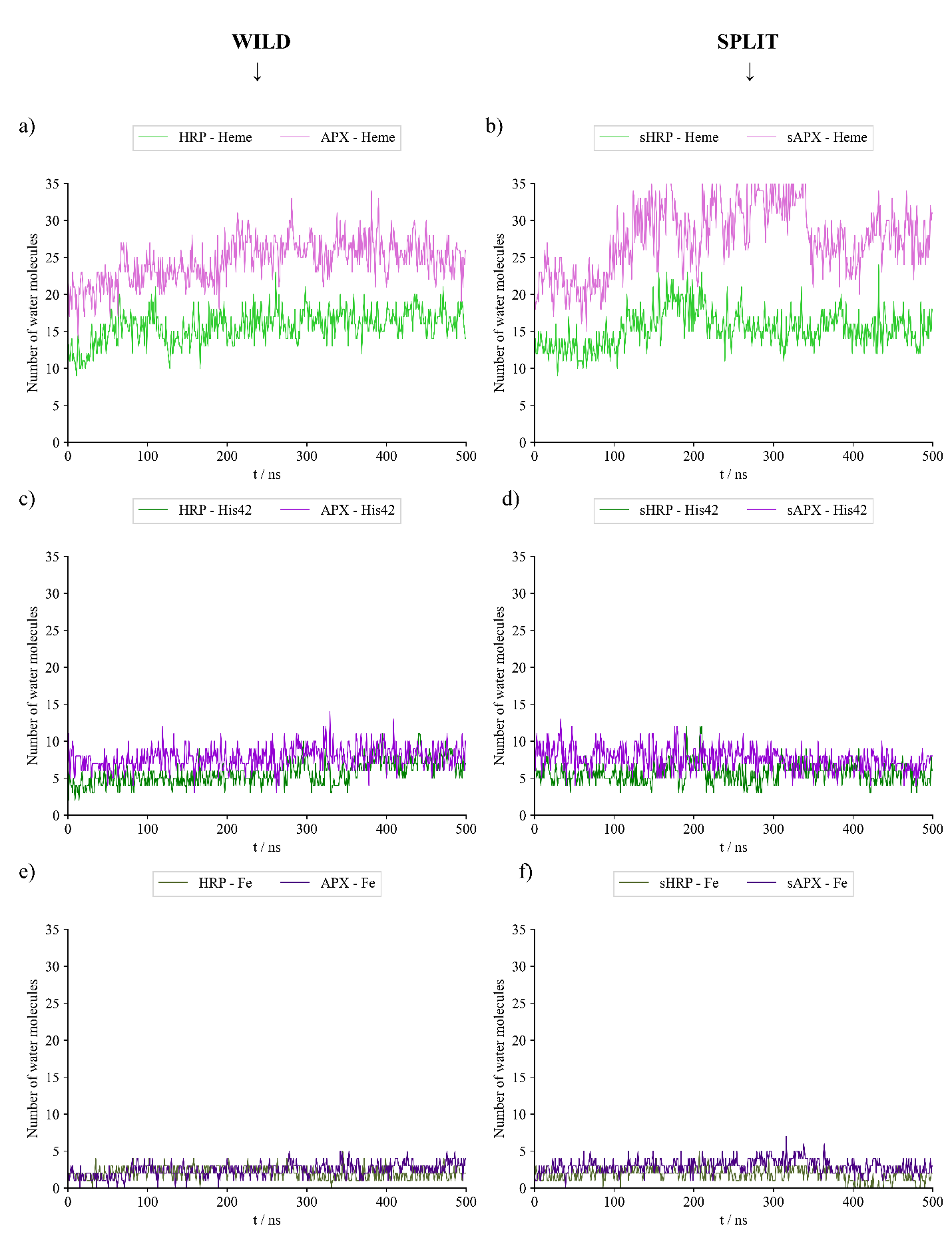


Figure S9. Number of water molecules within 0.5 nm form the the heme group, His42, and Fe^2+^ ion during 500 ns of MD simulation of: a) APX and HRP; b) sAPEX and sHRP.


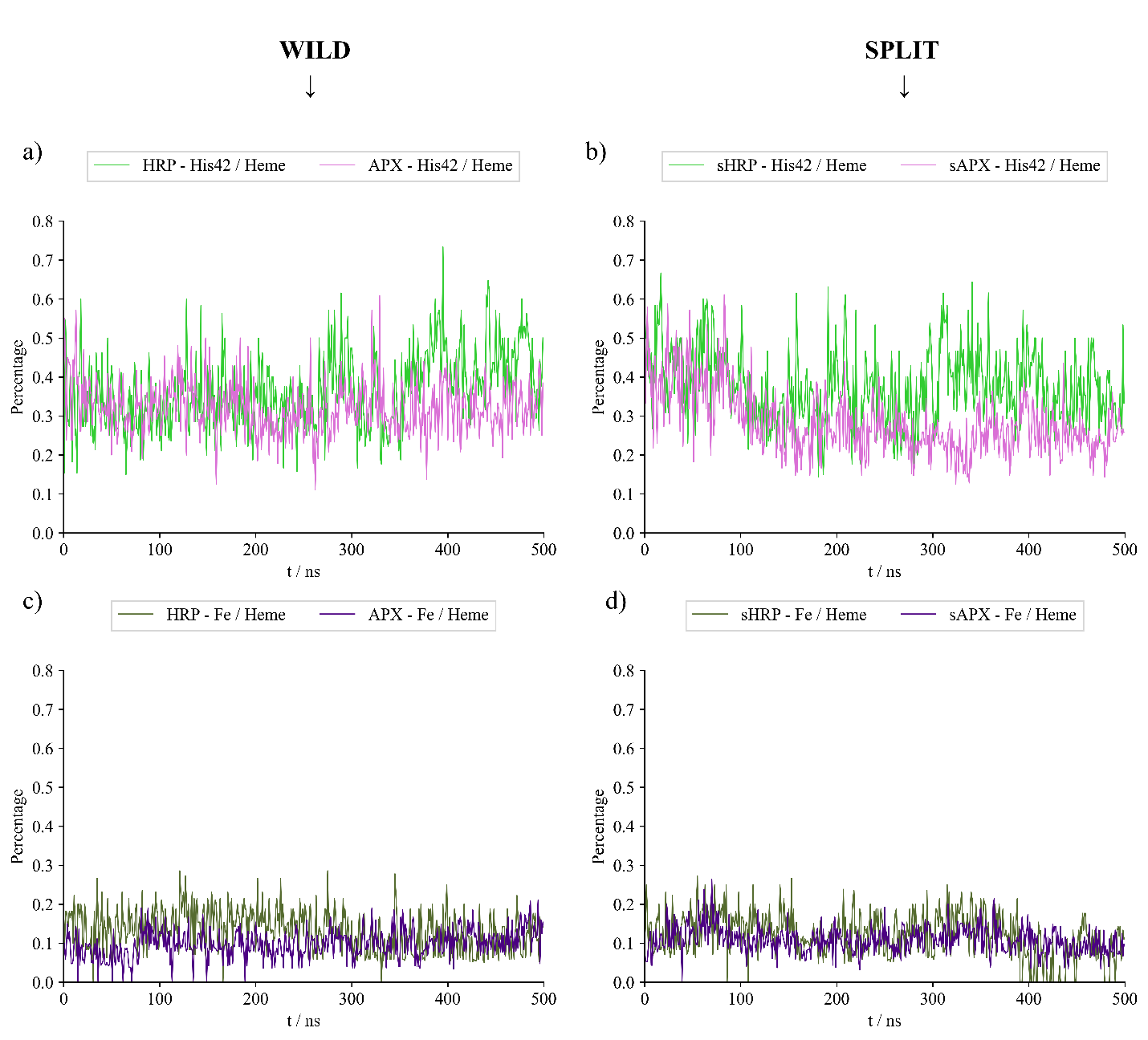


Figure S10: Ratio of water molecules that are 0.5 nm around His 42/heme and Fe/heme for: c) APX/HRP; d) sAPEX/sHRP.


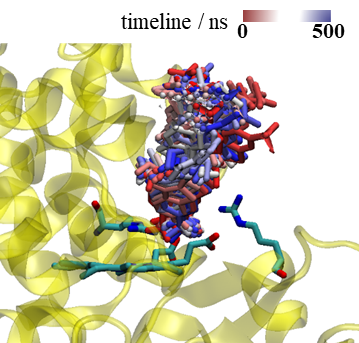


Figure S11: Timeline of aligned snapshots of ABTS substrate taken every 10 ns from trajectory in 500 ns simulation complex with active site of HRP protein. Snapshot of protein is taken on middle structure from the most populated cluster in last 200 ns of MD simulation. Hydrogen atoms are hidden for clarity.


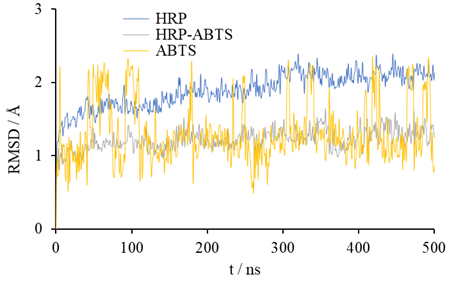


Figure S12: RMSD of HRP protein in simulation without complex, HRP protein in HRP-ABTS complex and ABTS substrate in HRP-ABTS complex.


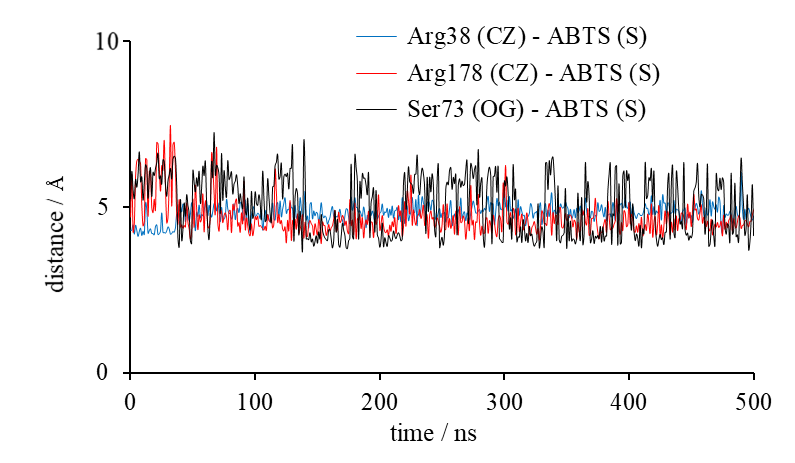


Figure S13: Distances in time of protein atoms CZ/OG of amino acids Arg38, Arg178 and Ser73 and substrate atom S (ABTS) in 500 ns simulation of HRP-ABTS complex.


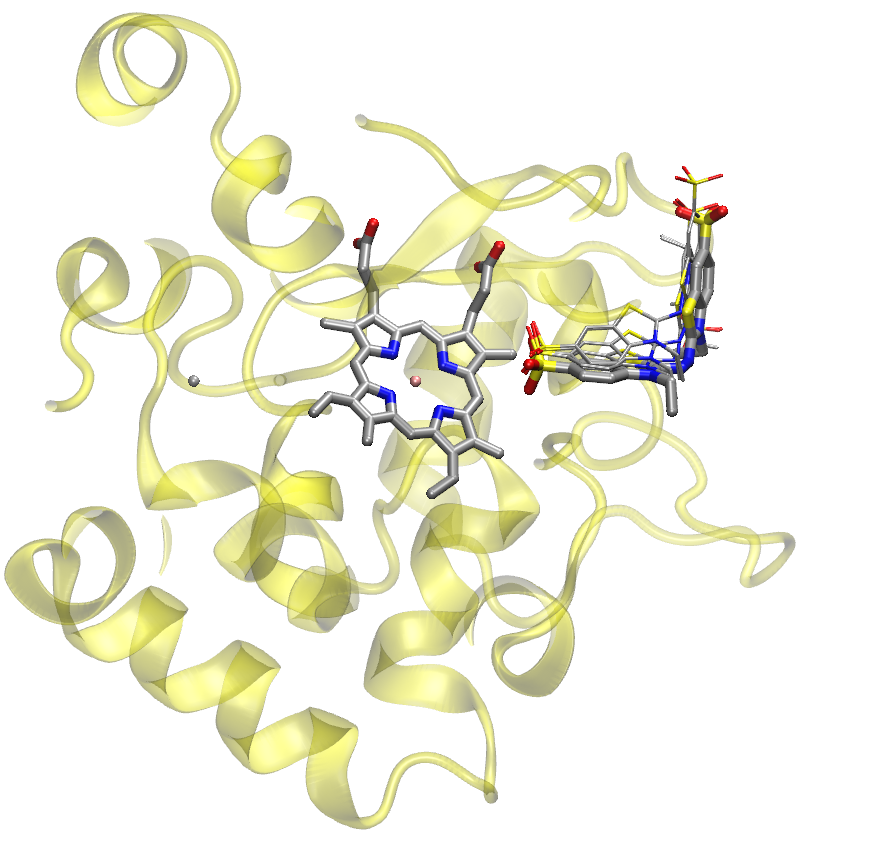


Figure S14: Docking results – all low energy binding modes are properly oriented for catalysis – 4 lowest energy binding modes of ABTS-HRP are depicted in thin lines. The starting structure for MD simulation of the HRP-ABTS complex was selected from the docking calculations performed on the equilibrated HRP protein structure (bolded ABTS structure).
